# Central obesity increases risk of breast cancer irrespective of menopausal and hormonal receptor status in women of South Asian Ethnicity

**DOI:** 10.1016/j.ejca.2016.07.022

**Published:** 2016-10

**Authors:** R. Nagrani, S. Mhatre, P. Rajaraman, I. Soerjomataram, P. Boffetta, S. Gupta, V. Parmar, R. Badwe, R. Dikshit

**Affiliations:** a Centre for Cancer Epidemiology, Tata Memorial Centre, Mumbai, 400 012, India; b Center for Global Health, U.S. National Cancer Institute, 9609 Medical Center Drive, Rockville, MD 20892-9760, USA; c Section of Cancer Surveillance, International Agency for Research on Cancer, 150 Cours Albert Thomas, 69372, Lyon CEDEX, France; d Institute For Translational Epidemiology, Mount Sinai Hospital, 1 Gustave L. Levy Place, New York, NY 10029-6574, USA; e Advanced Centre for Treatment, Research and Education in Cancer, Navi Mumbai, Maharashtra 400 012, India; f Department of Surgical Oncology, Tata Memorial Hospital, Mumbai, Maharashtra 400 012, India

**Keywords:** Breast cancer, Central obesity, Menopausal status, Hormone receptor status, South Asian

## Abstract

**Background:**

Current evidence suggests that the relationship between obesity and breast cancer (BC) risk may vary between ethnic groups.

**Methods:**

A total of 1633 BC cases and 1504 controls were enrolled in hospital-based case–control study in Mumbai, India, from 2009 to 2013. Along with detailed questionnaire, we collected anthropometric measurements on all participants. We used unconditional logistic regression models to estimate odds ratios (ORs) and 95% confidence interval (CI) for BC risk associated with anthropometry measurements, stratified on tumour subtype and menopausal status.

**Results:**

Waist-to-hip ratio (WHR) of ≥0.95 was strongly associated with risk of BC compared to WHR ≤0.84 in both premenopausal (OR = 4.3; 95% CI: 2.9–6.3) and postmenopausal women (OR = 3.4; 95% CI: 2.4–4.8) after adjustment for body mass index (BMI). Premenopausal women with a BMI ≥30 were at lower risk compared to women with normal BMI (OR = 0.5; 95% CI: 0.4–0.8). A similar protective effect was observed in women who were postmenopausal for <10 years (OR = 0.6; 95% CI: 0.4–0.9) but not in women who were postmenopausal for ≥10 years (OR = 1.8; 95% CI: 1.1–3.3). Overweight and obese women (BMI: 25–29.9 and ≥ 30 kg/m^2^, respectively) were at increased BC risk irrespective of menopausal status if their WHR ≥0.95. Central obesity (measured in terms of WC and WHR) increased the risk of both premenopausal and postmenopausal BCs irrespective of hormone receptor (HR) status.

**Conclusions:**

Central obesity appears to be a key risk factor for BC irrespective of menopausal or HR status in Indian women with no history of hormone replacement therapy.

## Highlights

• Waist-to-hip ratio increases risk of breast cancer (BC) irrespective of menopausal or hormone receptor status.
• High body mass index (BMI) protects the risk of BC in premenopausal women.
• The protection in risk of BC continues in women who have high BMI and are postmenopausal for <10 years.
• There is an increase in risk of BC in women have high BMI and are postmenopausal for ≥10 years.

## 1 Introduction

Recent trends have shown marked increase in breast cancer (BC) incidence in India, with a larger increase in postmenopausal compared to premenopausal women [1]. A potential explanation for this increase could be changing patterns of lifestyle factors as a result of rapid economic transition. In the last two decades, levels of physical activity have reduced, and food patterns have changed, leading to an increase in the average population body mass index (BMI) [2]. The prevalence of central obesity is particularly high in Indian population; and Indians are reported to have a higher body fat percentage than Caucasians for the same BMI [3].

Higher (≥30 kg/m^2^) BMI has been consistently associated with increased risk of postmenopausal BC [4] but decreased risk of premenopausal BC in Caucasian and Asian populations [5]. Central obesity has been associated with increased risk of BC in postmenopausal women [6], but its effect on premenopausal BC seems to vary according to ethnic status. Markers of central obesity such as waist-to-hip ratio (WHR) appear to show strong positive association for premenopausal Asian women, but smaller (increased risk of lower magnitude) for African and Caucasian women [6]. The studies from Asia too have largely been limited to Japan, China, Taiwan and Thailand [6].

We performed a case–control study at the Tata Memorial Hospital (TMH), Mumbai, India, to evaluate the risk of premenopausal and postmenopausal BC in relation to different measures of body fatness (BMI, WC and WHR) stratified on hormone receptor (HR) status in a population which has not been exposed to hormone replacement therapy and has not undergone systematic community screening for BC.

## 2 Material and methods

We conducted a hospital-based case–control study at TMH between January 2009 and September 2013. A total number of 1659 premenopausal (818 cases and 841 visitor controls) and 1478 postmenopausal women (815 cases and 663 visitor controls) were enrolled during the study period. The information on HR status, i.e., oestrogen receptor (ER), progesterone receptor (PR) and human epidermal growth factor receptor 2 (HER2) was available on 1294 (79.0%) BC cases. The premenopausal and postmenopausal BC cases were further stratified into oestrogen receptor positive/progesterone receptor positive (ER+/PR+), oestrogen receptor negative/progesterone receptor negative (ER–/PR–) and triple negative breast cancer (TNBC). The study has been approved by TMH Institutional Review Board.

### 2.1 Selection of cases

The cases were female BC patients coming to TMH. Only primary histologically confirmed BC cases aged 20–69 years were enrolled in the study with date of diagnosis not more than 6 months from the date of interview.

### 2.2 Selection of controls

All female visitors with no history of cancer coming along with any site cancer patient (e.g. breast, head and neck, thoracic, urology, gynaecology, etc) aged 20–69 years were included in the study. Controls were frequency matched to cases on age (±10 years) and region of residence (northern, western, central, southern and eastern India) at the time of enrolment. Eligible study participants were enrolled simultaneously during the study period. Forty percent of the controls enrolled in the study were first degree relatives (mother, sister or daughter) from various disease management groups (DMGs). The remaining were other relatives, friends and neighbours of different cancer site patients. The detail of questionnaire and study methodology has been mentioned in Supplementary Document.

### 2.3 Quality control

Data were checked at three levels (one by interviewer, study co-ordinator and data entry operator) and entered twice onto the software. We obtained over 90% correlation on all the variables collected on an abbreviated reproducibility questionnaire.

### 2.4 Exposure assessment

Menopausal status was assessed with the help of questionnaire. A woman was considered postmenopausal if the study participant responded that menstruation had stopped for more than 6 months at the time of interview. ER, PR and HER2 status were obtained from hospital pathology records.

**Fig. 1:**
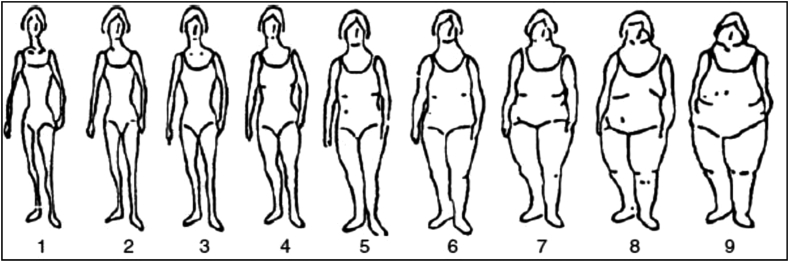
Pictogram for body size at different ages (10 years, 20 years and current). Nil.

The details of anthropometric measurements have been explained earlier [7]. WHR was calculated as waist circumference (WC) (in cm) divided by hip circumference (HC) (in cm) and grouped into three categories. BMI (kg/m^2^) was calculated by dividing weight in kilograms by the square of height (in m^2^). Postmenopausal women were divided into two groups for analysis of BMI: those who were postmenopausal for <10 years and those who were postmenopausal for ≥10 years at the time of enrolment. This stratification of postmenopausal women is in accordance with Pike *et al.* [8] who mentioned that menopausal transition shifts BMI from a protective factor to a risk factor of BC in almost a decade. We showed body size pictograms to all the study participants as depicted in Fig. 1 to mention their body sizes at three stages of life, i.e., age 10, 20 and at the time of enrolment. Body size pictograms at each stage were categorised into <3 (reference), 3–4, and ≥5 as per Fig. 1. Using the pictogram, increase in body size was estimated at two stages, i.e., from age 10 to 20 years and from age 20 to age at the time of enrolment. Each was categorised into no increase (reference), moderate increase and drastic increase. No increase was defined when the body size of the study participant remained between 1 and 2. Moderate increase was defined when the body size of the study participant increased from 1–2 to 3–4. Drastic increase was defined when the body size of the study participant increased from 1–2 to 5–9.

### 2.5 Statistical analysis

Odds ratios (ORs) and corresponding 95% confidence interval (CI) [9] were estimated for developing BC stratified on menopausal and HR status in relation to anthropometric factors. Unconditional logistic regression models were adjusted for potential confounders. To test for linear trends across quintiles, we assigned ordinal values to each quintile group and reported p_trend_ values. The sample size of 3000 (1500 cases and 1500 controls) was sufficient to detect an OR of 1.20 for risk factors having prevalence around 20% with 80% power based on assumed alpha level of 0.05. All analyses were performed using Stata version 12 [10].

## 3 Results

Study participants were enrolled from all regions of India with majority of participants residing in western parts of the country having 48.4% premenopausal and 51.7% postmenopausal BC cases (Table 1).

**Fig. 2:**
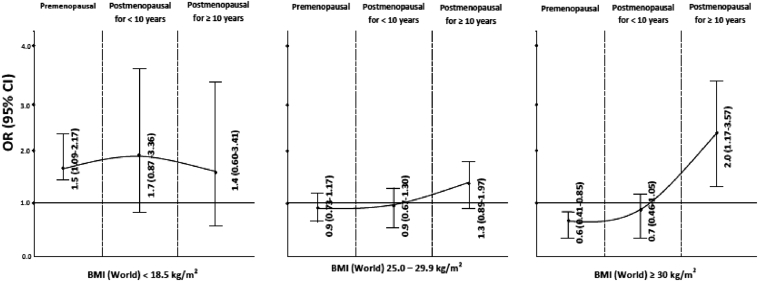
Relation of BMI and breast cancer stratified on menopausal status. BMI = 18.5–24.9—Reference. Adjusted for age, region of residence, rural-urban status, education, induced and spontaneous abortion, age at first full-term pregnancy. Details in Supplementary Table 1. BMI, body mass index; CI, confidence interval; OR, odds ratio.

The risk of developing BC in relation to BMI has been shown in Fig. 2. An increased risk of BC was observed for premenopausal and postmenopausal women with lower BMI (<18.5 kg/m^2^) compared to women with normal BMI (18.5–24.9 kg/m^2^), even after adjustment for WHR. BMI of ≥30 kg/m^2^ appeared to be protective for BC in premenopausal women compared to women with normal BMI with or without adjustment for WHR. When stratified by time of menopause, a decrease in BC risk (OR = 0.6; 95% CI: 0.39–0.91) was observed in women who were postmenopausal for <10 years, while BC risk increased in women with history of menopause for ≥10 years from enrolment in the study (OR = 1.8; 95% CI: 1.05–3.28), after adjustment for WHR (Supplementary Table 1).

Risks for developing BC in relation to various other anthropometric measurements and body size at different ages are tabulated in Table 2. Increased risk of BC with larger WC was observed in both premenopausal and postmenopausal women. WHR ≥0.95 was strongly associated with increased risk in both premenopausal (OR = 4.3; 95% CI: 2.90–6.31) and postmenopausal women (OR = 3.4; 95% CI: 2.39–4.79) compared to WHR ≤0.84 which remained statistically significant even after adjustment for WC (data not shown). Larger body size at age 20 years (≥5 versus <3) increased risk of premenopausal BC. Any increase in body size from age 10 to 20 years using pictogram was associated with increased risk in premenopausal BCs (OR = 1.4; 95% CI: 1.01–1.92).

Table 3 showed that WHR ≥0.95 increased risk of ER+/PR + BC (OR_pre_ = 3.71, 95% CI: 2.24–6.14; OR_post_ = 3.92, 95% CI: 2.45–6.27) and ER–/PR – BC (OR_pre_ = 5.41, 95% CI: 3.40–8.60; OR_post_ = 3.74, 95% CI: 2.40–5.81) compared to WHR ≤0.84.

**Fig. 3:**
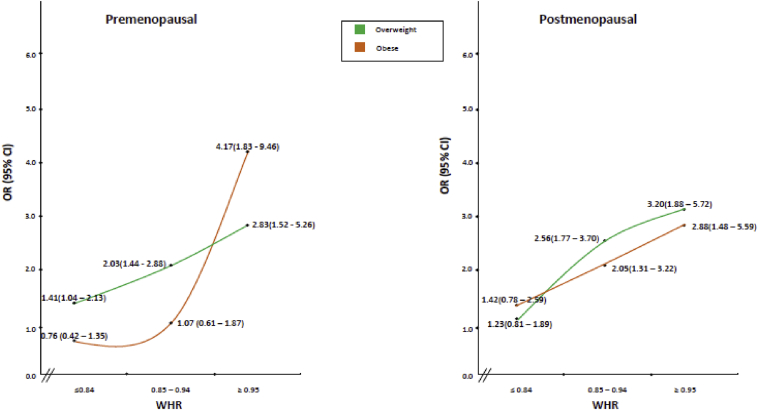
Relation of BMI and breast cancer stratified on menopausal status and waist-to-hip ratio. BMI = 18.5–24.9 and WHR = ≤0.84—Reference. Overweight: BMI = 25.0–29.9; obese: BMI = ≥30. Adjusted for age, region of residence, rural-urban status, education, induced and spontaneous abortion, age at first full-term pregnancy. Details in Supplementary Table 2. BMI, body mass index; CI, confidence interval; OR, odds ratio; WHR, waist-to-hip ratio.

Fig. 3 summarises the results of risk associated with BMI stratified on WHR for premenopausal and postmenopausal women. Increased risk for overweight and obese (in terms of BMI) women was observed in the highest category of WHR for both premenopausal and postmenopausal women (Supplementary Table 2).

## 4 Discussion

In the present study of obesity and BC in South Asian women, we found that high central obesity (measured by WHR) was most important risk factor, conferring an approximately threefold increased risk of BC. Increased BC risk with central obesity was observed for both premenopausal and postmenopausal women, even after adjustment for BMI, and even in women with BMI ≥30 kg/m^2^ in the highest category of WHR (Fig. 3). On further stratification by menopausal and HR status, the increased risk prevailed with central obesity in all strata irrespective of HR or menopausal status. Our results indicate that distribution of body fat, rather than BMI, is more important risk factor for BC in this Asian population.

The relationship between obesity and BC is complex, with different ethnic populations showing different patterns of risk depending on the particular measure of obesity [6]. These differences may be due to differences in body fatness (in terms of central obesity). It has been documented that ‘differences in body build and composition result in different relationship between BMI and body fat distribution in adult Asians relative to Caucasians’ [11]. Consistent with other studies [12], [13], we observed higher WC to be associated with an increased BC risk in both premenopausal and postmenopausal women.

Compared to other ethnic groups, Asian women have been previously reported to be associated with an increased BC risk associated with larger WHR (a measure of abdominal fat) among premenopausal and postmenopausal women [14], [15], although other studies have been inconclusive regarding ethnic differences [16], [17]. Our results support the hypothesis of a strong BC risk associated with central obesity in South Asian women, showing increased WHR-associated risk among both premenopausal (OR = 4.3; 95% CI: 2.9–6.1) and postmenopausal (OR = 3.4; 95% CI: 2.4–4.8) women after adjusting for BMI.

There have been inconsistencies with association of central obesity (measured in terms of WC and WHR) and BC when stratified on HR status [18], [19], [20]. Few studies have evaluated the association of WHR and WC with BC in relation to both menopausal and HR status [18], [21]. We observed an increase in risk of BC with an increase in central obesity in all tumour subtypes in both premenopausal and postmenopausal women (Table 3). This is consistent with the observations of John *et al.* [20] in premenopausal women and in other Asian populations [18]. Furthermore, the association of central obesity with increased insulin levels and insulin like growth factors may stimulate the growth of BC cells irrespective of ER/PR status [22].

Our results for BMI suggest a protective effect of higher BMI in premenopausal women, and an increased BC risk in women who were postmenopausal for ≥10 years even after adjusting for WHR. No increase in risk of BC was observed in women who were postmenopausal for <10 years which could possibly be due to carryover protective effect from premenopausal women. Pike *et al.* have argued that menopausal transition shifts BMI from a protective factor to a risk factor of BC in almost a decade. This effect was modelled to demonstrate that it takes a decade for a BMI of 30 kg/m^2^ in a premenopausal woman (at age 50 years, risk ratio [RR] of 0.75) to become a risk factor (RR of 1.20 at age 62 years) [8].

Given that a large proportion of women with normal BMI had a high WHR in our control population (Supplementary Table 2), BMI may not be a sensitive marker for obesity in this ethnic population. The increase in BC risk that we observed in overweight and obese women for premenopausal and postmenopausal women among the highest category of WHR suggests that the protection observed for higher BMI among premenopausal women might be because of higher muscle mass in younger women. Higher fat as reflected and probably more accurately measured by WC and WHR increases the risk for both premenopausal and postmenopausal BC.

Another interesting finding of the current study was observed association between low BMI and increased risk of BC irrespective of menopausal status. It is well known that even at low BMI, Indians are at higher risk of developing type 2 diabetes mellitus and metabolic syndrome [23], [24], [25]. Indians with low BMI have higher central obesity [3], [26], [27], [28]. Even in this study, 17.6% of controls with low BMI (<18.5 kg/m^2^) had higher WHR (≥0.85). Low BMI is associated with undernutrition and metabolic syndrome [29], [30]. The increased risk of low BMI observed in present study may thus be suggestive of risk related to metabolic syndrome. Although BC is not known to be associated with loss of weight, there may still be a possibility of reverse causality and this observation requires further replication in a population of similar background and ethnicity.

We found that height was positively associated with premenopausal BCs, which is consistent with previous reports [31], [32]. No such increase was observed for postmenopausal women, possibly due to low prevalence of taller women in the older cohort [33]. We observed an increase in risk of BC with increase in body size (using pictogram) from age 10 to 20 years for premenopausal women, but not for postmenopausal women, after adjusting for current BMI. Body size at age 20 years was associated with an increase in BC risk for premenopausal women (OR = 1.4; 95% CI: 1.03–1.83) and non-statistical significant increase for postmenopausal women when adjusted for current BMI.

Data on body size evolution and BC risk are limited; in contrast, an increase in weight has been associated with BC risk [34]. Most Indian women have low birth weight and higher weight at age 20 years (as indicated by pictogram). An increase in body size from age 10 to 20 years is thus indicative of accelerated growth in childhood which may result in increased adiposity and insulin resistance influencing BC risk. The self-reported current body size pictograms well correlated with measured BMI (r = 0.66) in present study. Previous studies have similarly used the pictograms to assess BC risk [31], [35]. However, we cannot completely exclude the possibility that women might have misrecalled their body size pictogram at different ages.

The controls were sampled from all DMGs and included all types of visitors (relatives, friends, neighbour, spouse, etc). As the sampling of controls was done from the same study base as cases and that non-responsiveness for study participants was less than 10%, we believe that findings of the study were not influenced by selection bias. To ensure quality of data and eliminate differential misclassification interviews were performed similarly in closed room by trained social workers with quality checking at three levels and 8% reproducibility for selected variables. Anthropometry measurements were performed twice by the same set of trained social workers for all study participants; thus, even if there was some misclassification, it would have been small and non-differential.

In conclusion, our study adds to the inconsistent literature on central obesity and risk of BC in Asian and particularly Indian women. We observed that higher WHR and WC were associated with threefold increased risk of premenopausal and postmenopausal BC. Future studies of BC should include more accurate measurements of body fatness and central obesity in particular (e.g. DEXA), possibly by incorporating measures of inflammatory markers, and focus on the role of nutrition and accelerated growth in teenagers as possible contenders for BC risk.

## Conflict of interest statement

None declared.

## Figures and Tables

**Table 1:** Summary characteristics of study participants.

Parameters	Categories	Premenopausal women (cases = 818; controls = 841)		Postmenopausal women (cases = 815, controls = 663)	
		Ca. (%)	Co. (%)	Ca. (%)	Co. (%)
Age at enrolment (years)	20–29	53 (6.4)	67 (7.9)	0	0
	30–39	340 (41.5)	353 (41.9)	16 (1.9)	10 (1.5)
	40–49	388 (47.4)	366 (43.5)	209 (25.6)	165 (24.8)
	50–59	37 (4.5)	54 (6.4)	401 (49.2)	342 (51.5)
	60–69	0	0	189 (23.1)	137 (20.6)
	Mean (±SD)	39.1 (±6.2)	38.45 (±6.8)	53.1 (±7.2)	53.2 (±6.9)
	Missing	0	1 (0.1)	0	9 (1.3)
Region of residence at enrolment	North	193 (23.5)	156 (18.5)	166 (20.3)	141 (21.2)
	West	372 (45.4)	432 (51.3)	422 (51.7)	343 (51.7)
	Central	51 (6.2)	43 (5.1)	46 (5.6)	42 (6.3)
	East	190 (23.2)	195 (23.1)	176 (21.6)	127 (19.1)
	South	12 (1.4)	15 (1.78)	5 (0.6)	10 (1.5)
	Missing	0	0	0	0
Education	No formal schooling	121 (14.7)	124 (14.7)	232 (28.4)	141 (21.2)
	<5 yrs of schooling	39 (4.7)	55 (6.5)	62 (7.6)	44 (6.6)
	5–8 yrs of schooling	183 (22.3)	200 (23.7)	177 (21.7)	162 (24.4)
	High school	247 (30.2)	271 (32.2)	204 (25.0)	181 (27.3)
	College graduation and more	227 (27.7)	189 (22.4)	138 (16.9)	134 (20.2)
	Missing	0.1	2 (0.2)	2 (0.2)	1 (0.1)
Age at menopause (years)	≤40	Not applicable		166 (20.3)	168 (25.3)
	41–45			223 (27.3)	181 (27.3)
	46–50			284 (34.8)	221 (33.3)
	>50			113 (13.8)	86 (12.9)
	Mean(±SD)			45.2 (±5.6)	44.7 (±5.9)
	Missing			29 (3.5)	7 (1.0)

Abbreviations: Ca, case; Co, control; SD, standard deviation.

**Table 2:** Association of anthropometric measurements, body size and breast cancer risk stratified by menopausal status.

Parameters	Categories	Premenopausal (cases = 818; controls = 841)					Postmenopausal (cases = 815, controls = 663)				
		Ca/Co	OR^a^ (95% CI)	p-value	OR^b^ (95% CI)	p-value	Ca/Co	OR^a^ (95% CI)	p-value	OR^b^ (95% CI)	p-value
Height^c^ (cm)	≤150	267/359	1.0 (ref)		1.0 (ref)		281/293	1.0 (ref)		1.0 (ref)	
	151–155	293/255	1.53 (1.22–1.94)	<0.001	1.77 (1.37–2.29)	<0.001	266/179	1.14 (0.89–1.45)	0.287	1.27 (0.97–1.65)	0.074
	156–160	164/175	1.24 (0.95–1.62)	0.102	1.43 (1.06–1.93)	0.019	117/137	0.65 (0.48–0.87)	0.004	0.71 (0.52–0.99)	0.047
	≥161	93/47	2.68 (1.82–3.95)	<0.001	3.03 (1.94–4.74)	<0.001	42/47	0.68 (0.43–1.06)	0.093	0.72 (0.44–1.19)	0.208
	P^d^			<0.001		<0.001			0.008		0.082
	P_heterogeneity_		0.0002								
Waist circumference (cm)	≤79	347/419	1.0 (ref)		1.0 (ref)		264/241	1.0 (ref)		1.0 (ref)	
	80–85	198/157	1.49 (1.16–1.93)	0.002	2.01 (1.49–2.71)	<0.001	133/131	0.92 (0.68–1.24)	0.611	1.27 (0.91–1.77)	0.158
	≥86	267/260	1.21 (0.96–1.52)	0.104	2.19 (1.58–3.04)	<0.001	410/284	1.31 (1.04–1.66)	0.020	2.40 (1.72–3.35)	<0.001
	P^d^			0.067		<0.001			0.015		<0.001
	P_heterogeneity_		0.018								
Waist-to-hip ratio	≤0.84	310/509	1.0 (ref)		1.0 (ref)		231/310	1.0 (ref)		1.0 (ref)	
	0.85–0.94	372/275	2.26 (1.82–2.80)	<0.001	2.43 (1.92–3.08)	<0.001	412/273	2.03 (1.61–2.55)	<0.001	2.33 (1.82–2.98)	<0.001
	≥0.95	130/52	4.28 (2.99–6.13)	<0.001	4.28 (2.90–6.31)	<0.001	163/73	3.02 (2.18–4.18)	<0.001	3.39 (2.39–4.79)	<0.001
	P^d^			<0.001		<0.001			<0.001		<0.001
	P_heterogeneity_		0.830								
Increase in body size from age 10 to 20 years (using pictogram)	No increase^e^	143/172	1.0 (ref)		1.0 (ref)		159/146	1.0 (ref)		1.0 (ref)	
	Any increase^f^	225/214	1.28 (0.95–1.71)	0.094	1.40 (1.01–1.92)	0.038	200/153	1.17 (0.86–1.60)	0.297	1.35 (0.97–1.88)	0.071
	Moderate increase^g^	177/155	1.40 (1.02–1.91)	0.034	1.54 (1.10–2.17)	0.012	151/116	1.18 (0.85–1.64)	0.317	1.29 (0.91–1.84)	0.145
	Drastic increase^h^	48/59	0.97 (0.62–1.52)	0.922	1.01 (0.62–1.65)	0.938	49/37	1.20 (0.74–1.94)	0.457	1.54 (0.91–2.60)	0.103
	P^d^			0.468		0.307			0.316		0.058
	P_heterogeneity_		0.180								
Increase in body size from age 20 to current age (using pictogram)	No increase^e^	29/28	1.0 (ref)		1.0 (ref)		26/22	1.0 (ref)		1.0 (ref)	
	Any increase^f^	135/165	0.76 (0.43–1.37)	0.376	1.16 (0.57–2.34)	0.669	147/132	0.97 (0.52–1.81)	0.941	1.57 (0.73–3.41)	0.246
	Moderate increase^g^	131/137	0.91 (0.51–1.63)	0.772	1.07 (0.58–2.00)	0.808	96/69	1.16 (0.60–2.22)	0.643	1.60 (0.79–3.25)	0.188
	Drastic increase^h^	199/211	0.87 (0.49–1.52)	0.628	1.25 (0.64–2.47)	0.503	224/202	0.90 (0.49–1.64)	0.736	1.33 (0.64–2.76)	0.430
	P^d^			0.596		0.402			0.308		0.894
	P_heterogeneity_		0.515								
Body size at age 10 years (using pictogram)	<3	372/389	1.0 (ref)		1.0 (ref)		360/302	1.0 (ref)		1.0 (ref)	
	3–4	247/253	1.03 (0.82–1.30)	0.739	1.12 (0.87–1.43)	0.359	225/176	1.07 (0.83–1.37)	0.581	1.07 (0.83–1.40)	0.568
	≥5	192/191	1.04 (0.81–1.33)	0.754	1.24 (0.95–1.62)	0.104	214/176	1.02 (0.79–1.31)	0.877	0.99 (0.76–1.30)	1.000
	P^d^			0.724		0.096			0.820		0.929
	P_heterogeneity_		0.594								
Body size at age 20 years (using pictogram)	<3	166/194	1.0 (ref)		1.0 (ref)		172/155	1.0 (ref)		1.0 (ref)	
	3–4	353/347	1.21 (0.94–1.57)	0.130	1.35 (1.02–1.78)	0.033	321/250	1.15 (0.88–1.52)	0.293	1.21 (0.91–1.61)	0.188
	≥5	285/284	1.17 (0.90–1.53)	0.235	1.37 (1.03–1.83)	0.028	302/241	1.12 (0.85–1.48)	0.386	1.20 (0.89–1.60)	0.214
	P^d^			0.305		0.042			0.451		0.677
	P_heterogeneity_		0.834								

Abbreviations: Ca/Co, cases/controls; CI, confidence interval; OR, odds ratio.

Missing values were excluded from analysis.

a Adjusted for age and region of residence.

b Adjusted for age, region of residence, rural-urban status, education, induced and spontaneous abortion, age at first full-term pregnancy, body mass index.

c Adjusted for current body weight and waist-to-hip ratio instead of body mass index.

d P for linear trend.

e No increase: body size (pictogram) remained between 1 and 2.

f Any increase: body size (pictogram) increased from 1–2 to 3–9.

g Moderate increase: body size (pictogram) increased from 1–2 to 3–4.

h Drastic increase: body size (pictogram) increased from 1–2 to 5–9.

**Table 3:** Association of BMI, WC and WHR and breast cancer risk stratified by menopausal and hormone receptor status.

Parameters	Categories	ER+/PR+ (cases = 569; controls = 1515)		ER–/PR– (cases = 725; controls = 1515)		TNBC (cases = 470; controls = 1515)	
		Ca/Co	OR^a^ (95% CI)	Ca/Co	OR^a^ (95% CI)	Ca/Co	OR^a^ (95% CI)
BMI in kg/m^2^ (premenopausal)^b^	<18.5	31/58	1.97 (1.18–3.30)	34/58	1.74 (1.06–2.87)	23/58	1.85 (1.05–3.26)
	18.5–24.9	160/400	1.0 (ref)	161/400	1.0 (ref)	103/400	1.0 (ref)
	25.0–29.9	91/270	0.69 (0.49–0.98)	118/270	0.92 (0.67–1.26)	87/270	1.04 (0.72–1.49)
	≥30	28/108	0.43 (0.25–0.73)	31/108	0.55 (0.34–0.89)	23/108	0.66 (0.38–1.16)
	P^c^		0.002		0.077		0.458
BMI in kg/m^2^ (postmenopausal for <10 years)^b^	<18.5	8/14	2.04 (0.72–5.77)	17/14	1.37 (0.60–3.13)	7/14	0.77 (0.26–2.31)
	18.5–24.9	49/157	1.0 (ref)	127/157	1.0 (ref)	83/157	1.0 (ref)
	25.0–29.9	68/141	1.59 (0.98–2.57)	69/141	0.60 (0.40–0.91)	40/141	0.53 (0.33–0.86)
	≥30	23/77	0.87 (0.47–1.61)	27/77	0.38 (0.22–0.66)	16/77	0.31 (0.15–0.61)
	P^c^		0.645		<0.001		<0.001
BMI in kg/m^2^ (postmenopausal for ≥10 years)^b^	<18.5	3/12	0.76 (0.15–3.76)	12/12	2.96 (1.11–7.91)	8/12	3.16 (1.00–9.98)
	18.5–24.9	35/129	1.0 (ref)	57/129	1.0 (ref)	35/129	1.0 (ref)
	25.0–29.9	39/99	1.36 (0.76–2.43)	40/99	0.91 (0.53–1.55)	25/99	0.88 (0.46–1.70)
	≥30	19/29	2.40 (1.11–5.16)	16/29	1.09 (0.51–2.32)	8/29	0.77 (0.29–2.08)
	P^c^		0.042		0.982		0.637
BMI in kg/m^2^ (all)^b^	<18.5	42/85	1.80 (1.17–2.78)	66/85	1.73 (1.17–2.54)	40/85	1.58 (1.01–2.47)
	18.5–24.9	248/690	1.0 (ref)	352/690	1.0 (ref)	223/690	1.0 (ref)
	25.0–29.9	201/513	0.98 (0.77–1.25)	229/513	0.80 (0.64–1.00)	154/513	0.82 (0.63–1.06)
	≥30	74/215	0.875 (0.54–1.05)	75/215	0.55 (0.40–0.77)	47/215	0.51 (0.35–0.76)
	P^c^		0.250		0.001		0.003
WC in cm (premenopausal)	≤79	140/419	1.0 (ref)	145/419	1.0 (ref)	94/419	1.0 (ref)
	80–85	72/157	1.67 (1.10–2.51)	86/157	2.15 (1.46–3.16)	64/157	2.47 (1.58–3.86)
	≥86	98/260	1.80 (1.15–2.80)	110/260	2.31 (1.52–3.51)	75/260	2.45 (1.49–4.01)
	P^c^		0.008		<0.001		<0.001
WC in cm (postmenopausal)	≤79	61/241	1.0 (ref)	143/241	1.0 (ref)	95/241	1.0 (ref)
	80–85	46/131	1.53 (0.94–2.49)	56/131	1.17 (0.77–1.80)	37/131	1.26 (0.77–2.08)
	≥86	148/284	2.52 (1.56–4.07)	174/284	2.71 (1.79–4.10)	96/284	2.42 (1.48–3.97)
	P^c^		<0.001		<0.001		<0.001
WC in cm (all)	≤79	201/666	1.0 (ref)	289/666	1.0 (ref)	190/666	1.0 (ref)
	80–85	118/288	1.57 (1.15–2.14)	144/288	1.66 (1.25–2.20)	101/288	1.85 (1.33–2.57)
	≥86	247/549	2.16 (1.57–2.97)	284/549	2.51 (1.88–3.36)	171/549	2.44 (1.72–3.45)
	P^c^		<0.001		<0.001		<0.001
WHR (premenopausal)	≤0.84	121/509	1.0 (ref)	125/509	1.0 (ref)	79/509	1.0 (ref)
	0.85–0.94	140/275	2.28 (1.65–3.16)	156/275	2.65 (1.95–3.60)	111/275	2.95 (2.06–4.22)
	≥0.95	49/52	3.71 (2.24–6.14)	60/52	5.41 (3.40–8.60)	43/52	6.20 (3.69–10.42)
	P^c^		<0.001		<0.001		<0.001
WHR (postmenopausal)	≤0.84	63/310	1.0 (ref)	104/310	1.0 (ref)	62/310	1.0 (ref)
	0.85–0.94	131/273	2.39 (1.66–3.45)	203/273	2.91 (2.12–4.01)	130/273	3.18 (2.16–4.67)
	≥0.95	61/73	3.92 (2.45–6.27)	65/73	3.74 (2.40–5.81)	36/73	3.83 (2.23–6.58)
	P^c^		<0.001		<0.001		<0.001
WHR (all)	≤0.84	184/825	1.0 (ref)	229/825	1.0 (ref)	141/825	1.0 (ref)
	0.85–0.94	272/550	2.31 (1.82–2.94)	361/550	2.78 (2.23–3.47)	241/550	3.10 (2.39–4.02)
	≥0.95	110/128	3.92 (2.80–5.49)	126/128	4.43 (3.23–6.08)	80/128	4.92 (3.39–7.12)
	P^c^		<0.001		<0.001		<0.001

Abbreviations: BMI, body mass index; Ca/Co, cases/controls; CI, confidence interval; ER, oestrogen receptor; OR, odds ratio; TNBC, triple negative breast cancer; WC, waist circumference; WHR, waist-to-hip ratio.

Missing values were excluded from analysis.

a Adjusted for age, region of residence, rural-urban status, education, induced and spontaneous abortion, age at first full-term pregnancy, body mass index.

b Adjusted for waist-to-hip ratio instead of body mass index.

c P for linear trend.
