# Supplementary material for: Central obesity increases risk of breast cancer irrespective of menopausal and hormonal receptor status in women of South Asian Ethnicity
Source: Eur J Cancer. 2016 Oct;66:153–61. doi: 10.1016/j.ejca.2016.07.022 (PMC5040194; doi:10.1016/j.ejca.2016.07.022)
Supplement: Supplementary file 1 [file mmc1.docx]

| **Supplementary Table 1: Association of BMI (kg/m^2^) – world and Risk of Breast Cancer stratified by Menopausal Status** | | | | | | | | |
| --- | --- | --- | --- | --- | --- | --- | --- | --- |
| **Parameters** | **Categories** | **Ca/Co** | **OR (95% CI)^a^** | **p-value** | **OR (95%CI)^b^** | **p-value** | **OR (95%CI)^c^** | **p-value** |
| Premenopausal | <18.5 | 81/58 | 1.40 (0.97-2.02) | 0.067 | 1.48 (1.009-2.17) | 0.045 | 1.82 (1.22-2.70) | 0.003 |
|  | 18.5-24.9 | 399/400 | 1.0 (ref) | | 1.0 (ref) | | 1.0 (ref) | |
|  | 25.0-29.9 | 261/270 | 0.93 (0.75-1.17) | 0.582 | 0.92 (0.73-1.17) | 0.537 | 0.85 (0.67-1.09) | 0.222 |
|  | ≥30 | 75/108 | 0.66 (0.47-0.91) | 0.014 | 0.59 (0.41-0.85) | 0.005 | 0.52 (0.36-0.76) | 0.001 |
|  | P^d^ | |  | 0.055 |  | 0.033 |  | 0.005 |
| Postmenopausal for < 10 years | <18.5 | 37/14 | 1.90 (0.99-3.66) | 0.052 | 1.71 (0.87-3.36) | 0.116 | 2.07 (1.04-4.14) | 0.038 |
|  | 18.5-24.9 | 221/157 | 1.0 (ref) | | 1.0 (ref) | | 1.0 (ref) | |
|  | 25.0-29.9 | 168/141 | 0.84 (0.62-1.14) | 0.275 | 0.94 (0.67-1.30) | 0.711 | 0.87 (0.62-1.22) | 0.432 |
|  | ≥30 | 72/77 | 0.65 (0.44-0.96) | 0.032 | 0.70 (0.46-1.05) | 0.085 | 0.60 (0.39-0.91) | 0.018 |
|  | P^d^ | |  | 0.029 |  | 0.144 |  | 0.037 |
| Postmenopausal for ≥ 10 years | <18.5 | 18/12 | 1.60 (0.74-3.49) | 0.229 | 1.44 (0.60-3.41) | 0.407 | 1.76 (0.72-4.26) | 0.21 |
|  | 18.5-24.9 | 117/129 | 1.0 (ref) | | 1.0 (ref) | | 1.0 (ref) | |
|  | 25.0-29.9 | 103/99 | 1.16 (0.80-1.69) | 0.423 | 1.33 (0.89-1.97) | 0.156 | 1.19 (0.79-1.80) | 0.382 |
|  | ≥30 | 48/29 | 1.90 (1.12-3.23) | 0.017 | 2.04 (1.17-3.57) | 0.012 | 1.85 (1.05-3.28) | 0.033 |
|  | P^d^ | |  | 0.044 |  | 0.014 |  | 0.057 |
| All postmenopausal women | <18.5 | 56/26 | 1.77 (1.08-2.90) | 0.022 | 1.60 (0.95-2.71) | 0.076 | 1.93 (1.12-3.30) | 0.016 |
|  | 18.5-24.9 | 347/286 | 1.0 (ref) | | 1.0 (ref) | | 1.0 (ref) | |
|  | 25.0-29.9 | 278/238 | 0.96 (0.76-1.21) | 0.738 | 1.07 (0.83-1.37) | 0.564 | 0.98 (0.76-1.26) | 0.889 |
|  | ≥30 | 125/106 | 0.97 (0.71-1.31) | 0.855 | 1.01 (0.74-1.39) | 0.913 | 0.89 (0.64-1.23) | 0.489 |
|  | P^d^ | |  | 0.673 |  | 0.748 |  | 0.566 |
| Abbreviations: BMI, Body Mass Index; CI, Confidence Interval; Ca/Co, Cases/Controls; OR, Odds Ratio.  ^a^ Adjusted for age and region of residence.  ^b^Adjusted on age, region of residence, rural-urban status, education, induced and spontaneous abortion, age at first full-term pregnancy.  ^c^ Adjusted on age, region of residence, rural-urban status, education, induced and spontaneous abortion, age at first full-term pregnancy, waist-to-hip ratio.  ^d^ P for linear trend  P _heterogeneity_ for BMI =0.450  Missing values were excluded from analysis. | | | | | | | | |

| **Supplementary Table 2: Association of BMI and Breast Cancer stratified on waist-to-hip ratio** | | | | | | | | | | | | |
| --- | --- | --- | --- | --- | --- | --- | --- | --- | --- | --- | --- | --- |
| **BMI (world)**  **category** | **Premenopausal** | | | | | | **Postmenopausal** | | | | | |
|  | **Waist-to-Hip ratio** | | | | | | | | | | | |
|  | **≤0.84** | | **0.85-0.94** | | **≥0.95** | | **≤0.84** | | **0.85-0.94** | | **≥0.95** | |
|  | **Ca/Co** | **OR^a^ (95% CI)** | **Ca/Co** | **OR^a^ (95% CI)** | **Ca/Co** | **OR^a^ (95% CI)** | **Ca/Co** | **OR^a^ (95% CI)** | **Ca/Co** | **OR^a^ (95% CI)** | **Ca/Co** | **OR^a^ (95% CI)** |
| <18.5 | 47/49 | 1.90 (1.19-3.02) | 28/9 | 6.68 (2.28-15.49) | 6/0 | No obs | 27/20 | 2.13 (1.08-4.20) | 24/6 | 4.94 (1.90-12.80) | 4/0 | No obs |
| 18.5-24.9 | 146/284 | 1.0 (ref) | 178/97 | 3.72 (2.64-5.25) | 69/19 | 5.76 (3.24-10.23) | 105/165 | 1.0 (ref) | 172/97 | 2.92 (2.02-4.21) | 72/23 | 5.17 (2.98-8.97) |
| 25.0-29.9 | 96/126 | 1.49 (1.04-2.13) | 133/122 | 2.03 (1.44-2.88) | 32/22 | 2.83 (1.52-5.26) | 68/90 | 1.23 (0.81-1.89) | 151/115 | 2.56 (1.77-3.70) | 58/32 | 3.20 (1.88-5.42) |
| ≥30 | 21/50 | 0.76 (0.42-1.35) | 30/47 | 1.07 (0.61-1.87) | 23/11 | 4.17 (1.83-9.46) | 30/34 | 1.42 (0.78-2.59) | 67/55 | 2.05 (1.31-3.22) | 28/17 | 2.88 (1.48-5.59) |
| Abbreviations: BMI, Body Mass Index; Ca/Co, Cases/Controls; CI, Confidence Interval; OR, Odds ratio.  ^a^ Adjusted for age, region of residence, rural-urban status, education, induced and spontaneous abortion, age at first full-term pregnancy. | | | | | | | | | | | | |
